# Hospital discharges for fever and neutropenia in pediatric cancer patients: United States, 2009

**DOI:** 10.1186/s12885-015-1413-8

**Published:** 2015-05-10

**Authors:** Emily L. Mueller, Kelly J. Walkovich, Rajen Mody, Achamyeleh Gebremariam, Matthew M. Davis

**Affiliations:** 1 Section of Pediatric Hematology Oncology, Department of Pediatrics, Indiana University School of Medicine, 410 West 10th Street, Suite 4099C, Indianapolis, IN 46202 USA; 2 Pediatric and Adolescent Comparative Effectiveness Research, Indiana University, Indianapolis, IN 46202 USA; 3 Division of Pediatric Hematology Oncology, Department of Pediatrics and Communicable Diseases, University of Michigan, Ann Arbor, MI 48109 USA; 4 Child Health Evaluation and Research (CHEAR) Unit, Division of General Pediatrics, Department of Pediatrics and Communicable Diseases, University of Michigan, Ann Arbor, MI 48109 USA; 5 Division of General Medicine, Department of Internal Medicine, University of Michigan, Ann Arbor, MI 48109 USA; 6 Institute for Healthcare Policy and Innovation, University of Michigan, Ann Arbor, MI 48109 USA; 7 Gerald R. Ford School of Public Policy, University of Michigan, Ann Arbor, MI 48109 USA

**Keywords:** Child, Adolescent, Oncology, Supportive care, Febrile neutropenia

## Abstract

**Background:**

Fever and neutropenia (FN) is a common complication of pediatric cancer treatment, but hospital utilization patterns for this condition are not well described.

**Methods:**

Data were analyzed from the Kids’ Inpatient Database (KID), an all-payer US hospital database, for 2009. Pediatric FN patients were identified using: age ≤19 years, urgent or emergent admit type, non-transferred, and a combination of ICD-9-CM codes for fever and neutropenia. Sampling weights were used to permit national inferences.

**Results:**

Pediatric cancer patients accounted for 1.5 % of pediatric hospital discharges in 2009 (n = 110,967), with 10.1 % of cancer-related discharges meeting FN criteria (n = 11,261). Two-fifths of FN discharges had a “short length of stay” (SLOS) of ≤3 days, which accounted for approximately $65.5 million in hospital charges. Upper respiratory infection (6.0 %) and acute otitis media (AOM) (3.7 %) were the most common infections associated with SLOS. Factors significantly associated with SLOS included living in the Midwest region (OR = 1.65, 1.22–2.24) or West region (OR 1.54, 1.11–2.14) versus Northeast, having a diagnosis of AOM (OR = 1.39, 1.03–1.87) or viral infection (OR = 1.63, 1.18–2.25) versus those without those comorbidities, and having a soft tissue sarcoma (OR = 1.47, 1.05–2.04), Hodgkin lymphoma (OR = 2.33, 1.62–3.35), or an ovarian/testicular tumor (OR = 1.76, 1.05–2.95) compared with patients without these diagnoses.

**Conclusion:**

FN represents a common precipitant for hospitalizations among pediatric cancer patients. SLOS admissions are rarely associated with serious infections, but contribute substantially to the burden of hospitalization for pediatric FN.

## Background

Fever and neutropenia (FN) is a common complication of chemotherapy for pediatric cancer patients [1]. FN is a well-recognized risk factor for morbidity and mortality in pediatric cancer populations [2], and the current standard of care is emergent broad-spectrum intravenous antibiotics and inpatient hospitalization until phagocyte recovery occurs [3, 4].

Despite the fact that hospitalizations for FN are common among pediatric cancer patients, recent data are limited regarding hospitalizations for pediatric FN at the national level. A recent analysis from 115 participating institutions in the United States (US) identified about 12,000 admissions for FN among pediatric cancer patients from 1995–2002, with a median length of stay (LOS) of 5 days [5]. No aggregate evaluation of hospitalizations for FN in pediatric cancer patients across the US has been published. A more comprehensive understanding of those patients with a short LOS may help inform future research aimed at decreasing the need for hospitalization of pediatric FN patients at low risk for serious complications.

The purpose of this study was to characterize discharges for pediatric cancer patients admitted non-electively and discharged with a diagnosis of fever and neutropenia, across the United States. We hypothesized that a substantial portion of pediatric cancer admissions for FN will have a short LOS, with few of these patients experiencing serious infections. Understanding this group of patient encounters could lead to interventions aimed at outpatient rather than inpatient management.

## Methods

### Study design and setting

Pediatric cancer patients admitted for FN were identified from a cross-sectional analysis of pediatric discharges in 2009, using the Healthcare Cost and Utilization Project’s (HCUP) Kids’ Inpatient Database (KID), compiled by the Agency for Healthcare Research and Quality (AHRQ) [6]. The KID is a nationally representative database that samples 80 % of pediatric discharges and 10 % of uncomplicated births at both teaching and non-teaching hospitals, compiled every third year since 1997; the most recent year of data available at the time of this project was 2009.

The KID is designed to increase the statistical power to detect and evaluate rare conditions among hospitalized children. The KID has been used in multiple instances to examine hospitalization patterns of care among children in the US [7–13]. Data elements within the KID include International Classification of Diseases, Ninth Revision, Clinical Modification (ICD-9-CM) codes, LOS, patient demographic characteristics, and hospital charges per episode of care.

Discharge weights based on the KID sampling scheme were applied to the data by AHRQ, to permit inferences regarding national patterns of hospital utilization by the authors. AHRQ provides information on the sampling design variables. HOSPID, KID_STRATUM and DISCWT are the cluster, the stratum, and the sample weight variables, respectively. We used survey estimation commands (SVY commands) in STATA incorporating the sample design variables. All data are presented as weighted values, unless otherwise specified. The 2009 KID data contained over 2 million unweighted discharges that represented over 7 million discharges in the population that year. Our analysis was based on de-identified national data and therefore was considered exempt from human subjects review by the University of Michigan Medical Institutional Review Board.

### Identification of cases

Using the KID, hospitalizations for pediatric cancer patients were determined using the HCUP Clinical Classification Software (CCS) for ICD-9-CM. The CCS is a diagnosis and procedure categorization scheme that aggregates ICD-9-CM codes into a smaller number of clinically meaningful categories. CCS codes 11–45 encompass all types of malignancies and therefore were used to define the pediatric cancer subpopulation. Transfers were excluded from all analyses for two reasons: (a) to avoid duplicate encounters for the same episode of care since patient identifiers are not included in this dataset; and (b) to accurately assess LOS, which would have been artificially shortened in the data of the transferring and receiving hospitals. We ascertained the proportion of children with FN who died while hospitalized; they were included in our analyses as a clinically important subgroup whose LOS was not affected by transfer.

### Outcome and explanatory variables

**Fig. 1:**
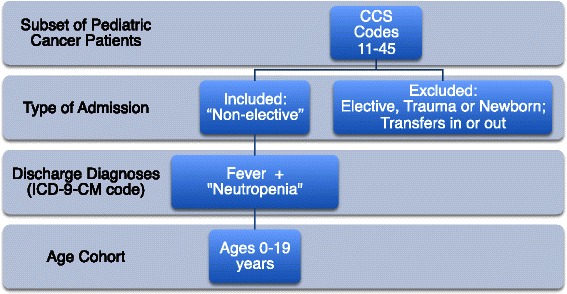
Case selection strategy for “Fever and Neutropenia” – Kids’ Inpatient Database for 2009

One of the aims of our study was to identify febrile neutropenic patient discharges associated with a relatively short, uneventful inpatient course and to assess variables associated with these discharges. Discharges with a short LOS would be potential candidates for future outpatient management strategies. Patients who develop fever while inpatient are traditionally considered high risk and would not qualify for early discharge or outpatient management strategies [14]. Therefore, to define a discharge encounter for “fever and neutropenia” (FN), the following criteria were applied: age ≤19 years, admit type either urgent or emergent (i.e., excluded elective admissions), and combination of ICD-9-CM discharge diagnosis of fever (780.6, 780.60, 780.61) and “neutropenia” (neutropenia [288.0], pancytopenia [284.1], or decreased white blood cell count [112.5]) in any of the discharge diagnosis fields (Fig. 1).

To identify co-occurring infections in patients with FN, ICD-9-CM codes were utilized. For common diagnoses to which multiple ICD-9-CM codes could map, general categories were defined, including pneumonia (480.x−485.x) and upper respiratory infection (460.x−465.x). Bloodstream infections were identified by the following ICD-9-CM codes: bacteremia (790.7), septicemia (38.x), infection due to a vascular device, implant or graft (996.62), or infection due to central venous line (999.31, 999.32).

Our primary outcome of interest was the frequency of discharges with a short LOS (SLOS), defined as ≤3 days in order to capture all hospitalizations that would have lasted 48 h or less. This threshold was selected because of the clinical relevance of negative blood cultures at 48 h [15], and because LOS is counted based on the number of midnights spent in the hospital [16]. For example, a child admitted at 11:59 pm on the 1^st^ of a month who had negative blood cultures at 11:59 pm on the 3^rd^ day of the month could be discharged on the 4^th^ day of the month (LOS = 3 days).

Secondary outcomes for FN discharges were related to population-adjusted frequency of discharges, mean LOS, and mean and total hospital charges per discharge for FN.

### Data Analyses

Descriptive statistics were used to demonstrate the distribution of FN discharges by gender, age, race/ethnicity, and primary payer type, hospital teaching and location status, and hospital Census region. The frequency of discharges for FN in pediatric oncology patients was described as the rate of discharges per 100,000 US children per year, using national Census data for 2009 [17]. Hospital charges were adjusted from 2009 US$ to 2014 US$ using the Consumer Price Index [18].

A weighted multivariate logistic regression model was used to estimate factors associated with SLOS for pediatric cancer patient encounters for FN; statistical weights also accounted for clustering of patients by hospital. Potential explanatory variables were selected for the model *a priori* based on hypothesized clinical relevance, which included: patient’s age, gender, primary expected payer, median household income for the patient’s zip code, hospital location and teaching status, hospital Census region, dichotomous variables for the presence or absence of each of the top 10 most common cancer diagnoses [19], and a dichotomous variables for types of infections. We hypothesized that sociodemographic and hospital level factors may influence admission and/or discharge decision making based on resources of the individual and within the community. Statistical analyses were performed using STATA version 13.0 (Stata Corp, College Station, TX).

## Results

### Characteristics of the study population

In 2009, there were a total of 7,370,203 weighted pediatric discharges in the U.S. Among those, 1.5 % (n = 110,967) were associated with a diagnosis of cancer. According to the HCUP classification, 49 % (n = 54,868) of pediatric cancer patients were admitted non-electively (coded as either urgent or emergent). Of all discharges for pediatric cancer patients, 10.1 % (n = 11,261) met criteria for FN; among non-elective pediatric cancer discharges, 19.3 % met criteria for FN. Only 1.2 % of pediatric cancer patient discharges ended in death—totaling 0.4 % among the pediatric FN discharges.

Characteristics of pediatric cancer patient discharges that met criteria for FN are presented in Table 1. The age distribution for FN discharges demonstrated the majority of patients are 0–9 years old, higher than the proportion among overall pediatric cancer admission for patients 0–9 years old. In addition, the study population was found to be predominately male and of non-Hispanic white race. Most FN patients were insured, with private insurance more common than public coverage. Just over one-half of the FN discharges had an associated diagnosis of acute lymphoblastic leukemia (ALL), versus 25 % of the overall pediatric cancer discharges with a diagnosis of ALL.

**Table 1:** Characteristics of discharges for non-transferred pediatric cancer patients: overall and for fever and neutropenia (FN) discharges – United States, 2009

	Proportion of overall pediatric cancer discharges	Proportion of pediatric FN discharges
	% (95 % CI)	
***Patient characteristics***		
Gender		
Female	45.0 (44.2–45.8)	46.5 (45.0–48.0)
Age		
0–4 years	28.9 (27.8–30.0)	36.7 (34.6–38.8)
5–9 years	22.0 (21.2–22.7)	27.9 (26.6–29.3)
10–14 years	21.3 (20.6–22.0)	18.2 (17.0–19.5)
15–19 years	27.8 (26.6–29.1)	17.2 (15.8–18.7)
Race/Ethnicity		
White	48.4 (44.2–52.7)	55.7 (50.5–60.9)
Black	9.7 (8.4–11.2)	7.1 (5.7–8.7)
Hispanic	21.2 (17.7–25.3)	17.1 (13.6–21.3)
Asian/pacific islander	7.9 (6.7–9.4)	7.6 (6.0–9.4)
Primary payer		
Public	39.2 (36.7–41.9)	37.1 (34.3–40.1)
Private	53.4 (50.9–55.9)	57.3 (54.1–60.4)
Self–pay	2.2 (1.5–3.1)	2.4 (1.4–3.9)
Other	5.2 (3.9–6.9)	3.2 (2.5–4.2)
Median household income per ZIP code		
1^st^ quartile	24.8 (22.5–27.3)	24.0 (20.7–27.8)
2^nd^ quartile	25.3 (50.9–55.9)	25.1 (23.0–27.2)
3^rd^ quartile	25.3 (1.5–3.1)	24.3 (22.2–26.6)
4^th^ quartile	24.6 (21.8–27.6)	26.5 (22.6–30.9)
Type of cancer		
ALL	24.6 (23.5–25.8)	44.3 (41.6–47.0)
Bone cancer	12.8 (12.0–13.6)	10.4 (9.1–12.0)
Central Nervous System tumor	9.6 (8.8–10.4)	6.2 (5.1–7.5)
AML	5.9 (5.5–6.3)	7.4 (5.9–9.1)
Soft tissue sarcoma	5.1 (4.7–5.6)	4.6 (3.9–5.4)
Neuroblastoma	4.5 (3.9–5.1)	4.7 (3.7–6.0)
Hodgkin lymphoma	3.2 (2.9–3.5)	2.9 (2.5–3.4)
Wilms tumor	2.7 (2.5–3.0)	3.0 (2.5–3.6)
Non–Hodgkin lymphoma	2.6 (2.3–2.9)	3.6 (3.1–4.3)
Ovarian or testicular tumor	1.7 (1.5–1.9)	0.7 (0.5–1.0)
***Hospital characteristics***		
Hospital Location-Teaching Status		
Rural	1.7 (1.0–2.8)	1.6 (0.7–3.3)
Urban, non-teaching	9.9 (7.0–13.8)	7.2 (4.4–11.5)
Urban, teaching	88.4 (84.4–91.5)	91.2 (86.7–94.3)
Hospital region		
Northeast	16.3 (11.2–23.0)	20.7 (13.6–30.2)
Midwest	21.5 (15.7–28.8)	26.0 (18.0–36.0)
South	36.3 (28.6–44.8)	40.2 (29.6–51.8)
West	25.9 (18.8–34.4)	13.1 (7.4–22.2)

In 2009, population-adjusted discharges for FN among pediatric cancer patients occurred at a rate of 13.4 per 100,000 US children. Over the study period, FN discharges in pediatric cancer patients represented 79,870 total inpatient days.

### Discharge characteristics for pediatric FN discharges

The distribution of LOS for FN discharges is included in Table 2, with SLOS admissions accounting for 41 % of all FN discharges. For patients with SLOS, the most commonly associated infection was an upper respiratory infection, followed by acute otitis media (Table 2). Bloodstream infection was present in 3.1 % of patients with SLOS, as compared to 20.8 % whose LOS lasted 8–14 days.

**Table 2:** Comparison of proportion of infectious diagnoses by LOS category among pediatric cancer FN discharges

	Overall	LOS category				
		“Short LOS”	4-7 days	8-14 days	15-30 days	31+ days
		≤3 days				
	Proportion (%)					
Proportion of FN DCs		41	33	16	7	3
No infection identified	75.9	82.7	77.3	66.9	62.7	44.4
Type of infection						
Upper respiratory infection	5.4	6.0	5.6	4.0	4.3	5.3
Acute otitis media	2.9	3.7	2.3	2.3	2.2	3.3
Bloodstream infection	10.4	3.1	9.5	20.8	23.9	35.6
Viral infection	2.3	3.1	2.3	1.6	0.2	0
Urinary tract infection	1.9	1.1	2.4	2.2	2.5	4.6
Pneumonia	1.2	0.3	0.6	2.3	4.1	6.8

### Charges for pediatric FN discharges

The overall mean hospital charge for a pediatric cancer patient discharge for FN was $52,160 (Table 3). As expected, mean charges varied substantially by LOS category. Given approximately 4,500 SLOS discharges for pediatric FN at the national level in a year, FN discharges with SLOS accounted for approximately $65.5 million in hospital charges.

**Table 3:** Mean charges for pediatric cancer fever and neutropenia discharges: overall and by length of stay category - United States, 2009

	Mean charges	Total charges
Overall	$52,160	$587,398,210
Length of stay category		
≤3 days – “Short LOS”	$14,549	$67,296,971
4–7 days	$33,423	$125,499,734
8–14 days	$72,552	$128,683,775
15–30 days	$166,316	$128,267,528
>30 days	$412,730	$138,805,796

Charges are adjusted to 2014 dollars using the Consumer Price Index adjustment

### Factors associated with short LOS for pediatric FN discharges

In a multivariate analysis (Table 4), factors associated with significantly increased odds of SLOS for FN included living in the Midwest region (OR = 1.65, 1.22–2.24) or the West region (OR=1.54, 1.11–2.14) of the US compared with the Northeast region. In addition, having a concomitant diagnosis of acute otitis media (OR=1.39, 1.03–1.87) or viral infection (OR=1.63, 1.18–2.25) was associated with SLOS, compared with patients without those diagnoses. In terms of oncologic diagnoses, soft tissue sarcoma (OR=1.25, 1.13–1.37), Hodgkin lymphoma (OR=1.66, 1.45–1.91), or ovarian/testicular tumors (OR=1.76, 1.05–2.95) were also associated with SLOS, compared with patients without these diagnoses.

**Table 4:** Multivariate logistic regression to evaluate factors associated with a “Short LOS” (≤3 days) among pediatric cancer fever and neutropenia discharges

Factors	Adjusted odds ratio (OR)	95% CI	P-value
Gender			
Female	1.06	1.02–1.10	0.006
Age			
15–19 years	Ref		
10–14 years	1.06	0.99–1.13	0.076
5–9 years	1.20	1.11–1.30	<0.001
0–4 years	1.08	1.01–1.15	0.029
Primary payer			
Public	Ref		
Private	1.08	1.02–1.13	0.004
Self-pay	1.05	0.89–1.23	0.591
Other	0.96	0.85–1.08	0.467
Median household income per ZIP code			
1^st^ quartile	Ref		
2^nd^ quartile	1.08	1.01–1.16	0.032
3^rd^ quartile	1.10	1.03–1.18	0.01
4^th^ quartile	1.07	1.00–1.15	0.05
Hospital location-teaching status			
Urban, teaching	Ref		
Urban, non-teaching	1.12	0.96–1.31	0.16
Rural	2.01	1.57–2.58	<0.001
Hospital region			
Northeast	Ref		
Midwest	1.22	1.09–1.36	<0.001
South	1.04	0.94–1.15	0.41
West	0.94	0.85–1.05	0.298
Type of infection			
Upper respiratory infection	1.07	0.97–1.18	0.165
Acute otitis media	1.26	1.10–1.44	0.001
Bloodstream infection	0.21	0.18–0.24	<0.001
Viral infection	1.82	1.55–2.15	<0.001
Urinary tract infection	0.44	0.39–0.50	<0.001
Pneumonia	0.27	0.22–0.32	<0.001
Type of cancer			
ALL	0.84	0.78–0.89	<0.001
Bone cancer	0.77	0.71–0.85	<0.001
Central nervous system tumor	0.99	0.92–1.07	0.787
AML	0.40	0.36–0.45	<0.001
Soft tissue sarcoma	1.25	1.13–1.37	<0.001
Neuroblastoma	0.56	0.48–0.66	<0.001
Hodgkin lymphoma	1.66	1.45–1.91	<0.001
Wilms tumor	0.59	0.52–0.68	<0.001
Non-Hodgkin lymphoma	0.46	0.39–0.54	<0.001
Ovarian or testicular tumor	0.63	0.52–0.76	<0.001

Controlled for Race; CI = Confidence Interval, ALL = Acute Lymphoblastic Leukemia; AML = Acute Myelogenous Leukemia; Type of Infection and Type of Cancer are presented as dichotomous variables

## Discussion

This study provides a recent, nationally representative analysis of inpatient hospital discharges for FN among pediatric cancer patients in the United States. Overall, among non-elective discharges that were neither transferred nor ended in patient death, we found that about 1 of every 5 pediatric cancer-related hospital discharges is related to FN. Of these, 41 % had a short LOS, which accounted for over $66 million in hospital charges in 2009 (expressed in 2014 US$). Patients with soft tissue sarcoma, Hodgkin lymphoma or ovarian/testicular tumors and those with a diagnosis of acute otitis media or viral infection had increased odds of having short LOS compared with their peers, which highlight potential opportunities to mitigate healthcare expenditures and improve quality of life for patients and their families through alternative management of pediatric FN in some cases.

The distribution of patient demographics found among all pediatric cancer patients in this study is consistent with the patterns observed in the SEER data regarding the same patient population [20]. When comparing the age distribution of FN patient encounters to the overall pediatric cancer population from our dataset, there are disproportionately more patients in the 0–9 years age group. Some factors contributing to younger patients suffering more FN events could be specific malignancy diagnoses and the associated intensity or duration of required treatment, type of central line catheter (Broviac catheter versus Port-a-Cath) [21], and exposures to infectious etiologies, particularly viruses [22]. This age-specific phenomenon merits further examination with more detailed patient-level data, to investigate factors related to increased risk for admission for FN.

Pediatric cancer patients represent a unique population of medically complex patients whose use of the health care system has not previously been well documented. For those who met criteria for FN, a substantial portion of patient encounters (41 %) had SLOS (≤3 days) with a small proportion of major infections documented. We believe that SLOS encounters indicate a potentially modifiable treatment process. There have been several studies surrounding the cost of inpatient versus outpatient care for FN, including a decision-analytic model for treatment options for pediatric FN which indicated that the most cost-effective therapy strategy was home-based parenteral therapy [23]. The present study represents the first analysis of the financial impact of hospitalization for pediatric FN in the US healthcare system at the national level, which demonstrates a large financial burden. In the current healthcare reform environment, it will be essential for future investigation of pediatric FN management to include comparisons of effectiveness and cost efficiencies related to alternative clinical management options. Analyses will need to be undertaken that include the perspectives of the healthcare system, pediatric oncology providers, and the patients and families who are all potentially affected by modifications in clinical management.

For pediatric FN patients with SLOS, the question remains if a risk stratification system or admission followed by step-down therapy approach, such as the ones implemented for adult patients with FN [24, 25], would decrease the number of patients requiring inpatient admission for FN. The feasibility, efficacy and/or safety of alternative therapies for FN in the pediatric population have been explored by other investigators. A recent systematic review and meta-analysis of 14 randomized, controlled trials of inpatient versus outpatient, and oral versus parenteral, management of pediatric low-risk FN showed no differences in efficacy or safety [26]. However, the authors recognized the limitations of the review to include that outcome measurements were not uniform among the studies included and therefore their analysis may not be generalizable. Despite initial attempts to risk stratify pediatric cancer patients [27], no consensus on risk stratification has been achieved [28], therefore hospitalization for all FN patients’ remains common practice.

While primary insurance and median household income per ZIP code were not found to be statistically significant factors, it is appreciated that they play an important role in the interaction of patients with the health care system. It is possible that differences in resource availability for certain families may influence their pre-hospital care and during-hospital circumstances. Alternate approaches by the hospital system or insurance plans could be implemented to offset the financial burdens for these patients; such as reimbursement for lodging near the hospital, travel, and meals which could be less costly than an inpatient admission. Patient and parent perspectives will be important to assess and address when implementing new strategies for the management of FN.

Another consideration is differences in findings across US Census regions, which raise questions about modifications of care that may be more applicable to some regions than others. Factors such as ease of access and distance to patients’ cancer treatment centers, ability of local emergency departments to provide adequate care for pediatric cancer patients, and other system-wide practices, policies, and coordination at the state and regional levels could greatly affect the logistics of altering current standard practices.

Pediatric cancer patients with FN are routinely admitted to empirically treat and evaluate for a serious bacterial infection. Our investigation revealed that the rate of bloodstream infections among all pediatric cancer patient encounters with FN was 10.4 %. This was consistent with the lower limit of previous analyses that revealed bacteremia rates among children with cancer to be between 10 to 24 % [29–31]. As expected, the proportion of bloodstream infections was higher for those patients with longer LOS, likely representing a higher severity of illness among those with long LOS. Conversely, the proportion of SLOS discharges with a bloodstream infection was lower, at 3.1 %. A future analysis with patient-level data would better characterize whether patients with SLOS have reasons other than empiric antibiotics and monitoring that are necessitating hospitalization. Interestingly, a recent analysis found the time to positive blood cultures in febrile neonates was <24 h in over 90 % of cases [32]. A similar analysis among pediatric cancer patients could aid in decision-making for early discharge with less inpatient observation time required.

### Limitations

As an analysis of national administrative data, our study has many strengths but also notable limitations. Administrative data must rely on accuracy of ICD-9-CM codes to identify the population of interest, which are subject to errors of omission or commission. We did not have information regarding the cause for admission and therefore inferred the reason based on discharge diagnoses. FN is not a single diagnostic code and was indicated by the presence of both ICD-9-CM codes for fever and neutropenia. It is possible that this was an underestimation of patients with FN if some patients who present with fever and neutropenia are only discharged with the diagnosis of neutropenia. In addition, viral infection and upper respiratory infection are separate ICD-9-CM codes, but likely represent a spectrum of overlapping diseases that cannot be fully differentiated using this database. Variation in administrative coding and clinical management may exist between regions in the US. Importantly, administrative databases do not hold detailed information regarding the patients’ cancer staging, therapy regimens, vital signs or laboratory values, which are all important factors in the decision making regarding admission for a patient experiencing FN [15, 24].

## Conclusion

Our study demonstrates the substantial impact on healthcare utilization by pediatric cancer patients with discharges for FN, in a nationally representative dataset. Previous studies have attempted to determine the feasibility and safety of alternative approaches to clinical management of pediatric FN, yet these practices have not been widely accepted or adopted. Identification of the burden of FN is a key step in the process of developing awareness and motivation for a multi-center or national study to improve care of pediatric cancer patients experiencing FN.

## Abbreviations

AOM: Acute otitis media
AHRQ: Agency for Healthcare Research & Quality
CCS: Clinical Classification Software
ED: Emergency department
FN: Fever and neutropenia
KID: Kids’ Inpatient Database
ICD-9-CM: International Classification of Diseases, ninth revision, Clinical Modification
LOS: Length of stay
SLOS: Short length of stay
US: United States
