# Correction to: Hospital discharges for fever and neutropenia in pediatric cancer patients: United States, 2009

**DOI:** 10.1186/s12885-017-3654-1

**Published:** 2017-10-02

**Authors:** Emily L. Mueller, Kelly J. Walkovich, Rajen Mody, Achamyeleh Gebremariam, Matthew M. Davis

**Affiliations:** 1 Section of PediatricHematology Oncology, Department of Pediatrics, Indiana University School of Medicine, 410 West 10th Street, Suite 4099C, Indianapolis, IN 46202 USA; 2 Pediatric and Adolescent Comparative Effectiveness Research, Indiana University, Indianapolis, IN 46202 USA; 3 Division of Pediatric Hematology Oncology, Department of Pediatrics and Communicable Diseases, University of Michigan, Ann Arbor, MI 48109 USA; 4 Child Health Evaluation and Research (CHEAR) Unit, Division of General Pediatrics, Department of Pediatrics and Communicable Diseases, University of Michigan, Ann Arbor, MI 48109 USA; 5 Division of General Medicine, Department of Internal Medicine, University of Michigan, Ann Arbor, MI 48109 USA; 6 Institute for Healthcare Policy and Innovation, University of Michigan, Ann Arbor, MI 48109 USA; 7 Gerald R. Ford School of Public Policy, University of Michigan, Ann Arbor, MI 48109 USA

## Correction

After publication of the original article [1] the authors found that the following errors had occurred:

1. There was an error in the ICD-9-CM coding present in the manuscript. Specifically, the variable for “decreased white blood cell count” was incorrectly coded. The “decreased white blood cell count” variable accounted for a small proportion of the total population studied. There were only 11 fewer patients in the final fever and neutropenia population.
  Reanalyzing the data using the corrected ICD-9-CM codes yielded nearly identical results to those found in the original manuscript. None of the slight changes to odds ratios and *p*-values changed the inferences of the results, except for overall charges for short length of stay (3 days or less) discharges decreased from $65.5 to $60.5 million US dollars.
  The affected values in Tables 1, 2 and 3 from the original manuscript have been amended to reflect these small changes to estimates and *p*-values and are included in this Correction.
2. The original manuscript includes an incorrect version of Table 4. The estimates presented in the results section of the manuscript are accurate, as is the discussion of these results throughout the text and abstract. The revised Table 4 is included in this Correction and has also been adjusted to take into account the above-mentioned error in coding.

**Table 1:** Characteristics of Discharges for Non-Transferred Pediatric Cancer Patients: Overall and for Fever and Neutropenia (FN) Discharges – United States, 2009

	Proportion of Overall Pediatric Cancer Discharges	Proportion of Pediatric FN Discharges
	% (95% CI)	
***Patient Characteristics***		
Gender		
Female	45.0 (44.2–45.8)	46.5 (45.0–48.0)
Age		
0–4 years	28.9 (27.8–30.0)	36.7 (34.6–38.8)
5–9 years	22.0 (21.2–22.7)	27.9 (26.6–29.3)
10–14 years	21.3 (20.6–22.0)	18.2 (17.0–19.6)
15–19 years	27.8 (26.6–29.1)	17.1 (15.7–18.7)
Race/Ethnicity		
White	48.4 (44.2–52.7)	55.7 (50.5–60.8)
Black	9.7 (8.4–11.2)	7.1 (5.7–8.7)
Hispanic	21.2 (17.7–25.3)	17.1 (13.6–21.3)
Asian/Pacific Islander	7.9 (6.7–9.4)	7.5 (6.0–9.4)
Primary Payer		
Public	39.2 (36.7–41.9)	37.1 (34.2–40.0)
Private	53.4 (50.9–55.9)	57.3 (54.1–60.4)
Self-pay	2.2 (1.5–3.1)	2.4 (1.4–4.0)
Other	5.2 (3.9–6.9)	3.2 (2.5–4.2)
Mean Household Income per Zip Code		
1st quartile	24.8 (22.5–27.3)	24.1 (20.7–27.8)
2nd quartile	25.3 (50.9–55.9)	25.1 (23.0–27.2)
3rd quartile	25.3 (1.5–3.1)	24.3 (22.2–26.6)
4th quartile	24.6 (21.8–27.6)	26.5 (22.6–30.8)
Type of Cancer		
ALL	24.6 (23.5–25.8)	44.3 (41.6–47.0)
Bone Cancer	12.8 (12.0–13.6)	10.4 (9.1–12.0)
Central Nervous System Tumor	9.6 (8.8–10.4)	6.2 (5.1–7.6)
AML	5.9 (5.5–6.3)	7.4 (5.9–9.1)
Soft Tissue Sarcoma	5.1 (4.7–5.6)	4.6 (3.9–5.4)
Neuroblastoma	4.5 (3.9–5.1)	4.7 (3.7–6.0)
Hodgkin Lymphoma	3.2 (2.9–3.5)	2.9 (2.5–3.4)
Wilms Tumor	2.7 (2.5–3.0)	3.0 (2.5–3.4)
Non-Hodgkin Lymphoma	2.6 (2.3–2.9)	3.6 (3.1–4.3)
Ovarian or Testicular Tumor	1.7 (1.5–1.9)	0.7 (0.5–1.0)
***Hospital Characteristics***		
Hospital Location-Teaching Status		
Rural	1.7 (1.0–2.8)	1.6 (0.7–3.3)
Urban, non-teaching	9.9 (7.0–13.8)	7.2 (4.4–11.6)
Urban, teaching	88.4 (84.4–91.5)	91.2 (86.7–94.3)
Hospital Region		
Northeast	16.3 (11.2–23.0)	20.7 (13.6–30.2)
Midwest	21.5 (15.7–28.8)	26.0 (18.0–36.0)
South	36.3 (28.6–44.8)	40.2 (29.7–51.8)
West	25.9 (18.8–34.4)	13.1 (7.4–22.2)

**Table 2:** Comparison of Proportion of Infectious Diagnoses by LOS Category Among Pediatric Cancer FN Discharges

	Overall	LOS Category				
		“Short LOS” ≤3 days	4–7 days	8–14 days	15–30 days	31+ days
Proportion (%)						
Proportion of FN DCs		41	33	16	7	3
No Infection Identified	75.9	82.7	77.3	66.9	62.8	44.4
Type of Infection						
Upper Respiratory Infection	5.4	6.0	5.6	4.0	4.1	5.3
Acute Otitis Media	2.9	3.7	2.3	2.3	2.2	3.3
Bloodstream Infection	10.4	3.1	9.5	20.8	23.8	35.6
Viral Infection	2.3	3.1	2.3	1.6	0.2	0
Urinary Tract Infection	1.9	1.1	2.3	2.2	2.6	4.6
Pneumonia	1.1	0.3	0.6	2.3	4.1	6.8

**Table 3:** Mean Charges for Pediatric Cancer Fever and Neutropenia Discharges: Overall and by Length of Stay Category - United States, 2009

	Mean Charges	Total Charges
Overall	$46,938	$528,052,500
Length of Stay Category		
≤ 3 days – “Short LOS”	$13,098	$60,538,956
4–7 days	$30,092	$112,935,276
8–14 days	$65,313	$115,604,010
15–30 days	$149,780	$115,031,040
> 30 days	$371,506	$124,826,016

Charges are adjusted to 2014 dollars using the Consumer Price Index adjustment

**Table 4:** Multivariate Logistic Regression to Evaluate Factors Associated with a “Short LOS” (≤3 days) Among Pediatric Cancer Fever and Neutropenia Discharges

Factors	Adjusted Odds Ratio (OR)	95% CI	*p*-value
Gender			
Female	1.00	0.88–1.12	0.943
Age			
15–19 years	Ref		
10–14 years	0.98	0.78–1.23	0.873
5–9 years	1.23	0.98–1.54	0.071
0–4 years	1.15	0.95–1.40	0.148
Primary Payer			
Public	Ref		
Private	1.14	0.99–1.31	0.065
Self-pay	1.18	0.71–1.95	0.533
Other	1.30	0.93–1.82	0.125
Median Household Income per Zip Code			
1st quartile	Ref		
2nd quartile	1.09	0.89–1.33	0.390
3rd quartile	1.09	0.91–1.31	0.334
4th quartile	1.11	0.9–1.37	0.316
Hospital Location-Teaching Status			
Urban, teaching	Ref		
Urban, non-teaching	0.76	0.55–1.06	0.110
Rural	1.23	0.80–1.91	0.346
Hospital Region			
Northeast	Ref		
Midwest	1.66	1.23–2.24	0.001
South	1.25	0.99–1.57	0.062
West	1.54	1.11–2.15	0.01
Type of Infection			
Upper Respiratory Infection	1.12	0.90–1.40	0.294
Acute Otitis Media	1.39	1.03–1.87	0.031
Bloodstream Infection	0.17	0.14–0.22	<0.001
Viral Infection	1.63	1.18–2.25	0.003
Urinary Tract Infection	0.50	0.32–0.76	0.001
Pneumonia	0.28	0.13–0.60	0.001
Type of Cancer			
ALL	0.94	0.77–1.13	0.492
Bone Cancer	1.24	0.98–1.58	0.075
Central Nervous System Tumor	1.06	0.81–1.39	0.651
AML	0.35	0.25–0.49	<0.001
Soft Tissue Sarcoma	1.46	1.05–2.04	0.024
Neuroblastoma	0.65	0.46–0.93	0.019
Hodgkin Lymphoma	2.32	1.61–3.35	<0.001
Wilms Tumor	1.26	0.84–1.89	0.260
Non-Hodgkin Lymphoma	0.73	0.52–1.01	0.061
Ovarian or Testicular Tumor	1.76	1.05–2.95	0.031

## Corrected Tables

 Corrected Table 1

 Corrected Table 2

 Corrected Table 3

 Corrected Table 4
